# The Therapeutical Uses of the Muriate of Ammonia

**Published:** 1861-12

**Authors:** M. O. Heydock


ARTICLE II.

### THE THERAPEUTICAL USES OF THE MURIATE OF AMMONIA.

By M. O. HEYDOCK, M. D.

A Paper read before the CHICAGO MEDICAL SOCIETY.

I have thought that a paper, which should embody all the written experience which a hasty examination of my library and the journals within my reach furnishes, in reference to the valuable qualities possessed by a drug, little used in this country and England—might not prove uninteresting.

I will, therefore, give you a synopsis of the written experience of a few of our brethren, in the *Use of the Muriate, or Hydrochlorate of Ammonia*, my own coinciding with theirs, as far as I have had an opportunity of observing.

It is a matter of surprise with those who have used this drug, why it is so neglected by the profession, while a hundred articles of the Materia Medica, whose value and power are matters of doubt and scepticism with many of the faculty, especially those articles, which, for want of a more exact nomenclature, we give a place under the unsatisfactory appellation of Alteratives and Deobstruents—enter into our prescriptions, conjoined with medicines, whose active properties are satisfactorily proven.

Sarsaparilla has had its ups and downs; but I think it is conclusively settled by an impartial and rigid series of tests, to which it has been subjected, that it is a medicine of little alue, but an expensive vehicle for some whose merits cannot be called in question. And yet this article has a position in the popular estimation as a cure-all, second to none, and judging from prescriptions, some of the fraternity will take issue with me upon its merits. Yet, I can but think it a negative, rather than a positive medicine. Contrasted with the popularity of this expensive drug, the muriate of ammonia, so valuable, and at the same time, so cheap, has failed to win our confidence, only because we have never given it an opportunity, certainly, to the extent its virtues merit. In Germany, its position is much like that held by nitrate of potash with us. I am told that our German brethren in this city, use it very extensively. We all admit that it has few superiors as a refrigerant, externally—a Mr. Walker, according to Pariera, finding that a mixture composed of mur. ammonia and nitrate of potash *aa.* five parts, with sixteen parts of water, lowered the themometer from 50° to 10° F.

Let us now see how it stands in the estimation of a few, who, having used it, have called the attention of the profession to it:

One writer considers it " the most valuable of all the preparations of ammonia."

Pariera says it is liquefacient, resolvent and alterative ; promoting the mucous and cutaneous secretions, and under certain circumstances, that of menstruation ; counteracting organic abnormal conditions, as tumors, and thickening of mucous and serous membranes. Its diuretic effects are less obvious. It acts in many respects like the preparations of mercury, and yet a prolonged use of it does not give rise to the cachexia which results from the latter. In large doses, it is said to purge, in small ones it constipates. Its effects are much like iodine, bromine, and chlorine.

Dr. Lindsay, in the Glasgow *Medical Journal*, of 1855, gives an interesting paper, containing the results of his own experience with this drug upon himself and two friends. The condition of the system was observed as to appetite ; pulse, intestinal and urinary secretions, and articles of diet consumed. One took 9 grs., another 13½, and the last 18 grs.

per day, in three divided doses. No comparisons were instituted until at the close of the week

Upon the second day of trial, great buoyancy of the system was experienced, and an inclination for increased exertion in all three, representing the different temperaments, least in the lymphatic,—but it should also be stated that he consumed less of the drug than either of the others, taking but 9 grs.; with him the appetite was much increased, the amount of food doubled, the feculent discharges much augmented, the mucous follicles of the intestines seemed to be stimulated to much increased secretion. In two, the force and frequency of the heart was diminished, but the pulse slightly increased in the case of the smallest quantity. The urine was increased in two, from six to ten ounces, in the twenty-four hours, the excess of solids varying from 70 to 160 grs. per day. In the medium dose, the bowels seemed to be affected rather than the kidneys, so that it can hardly be called a renal hydragogue.

He has used it only in chronic diseases, such as are the results of inflammatory action, of which chronic bronchitis, enlargement of lymphatic glands, either scrofulous or of a syphilitic character, chronic skin diseases, and rheumatism, are the representatives.

He thinks its action much like iodide of potassium, and says that long continued use of it does not derange the digestive organs, like the alkalies. He give the details of treatment in two or three interesting cases of chronic bronchitis, attended with thick tenacious secretion, where the benefit was very marked, and also a case of constitutional syphilis where iod. potass. having been used in vain, the ammonia was resorted to with complete success.

Dr. Rea, in the London *Lancet*, of February, 1859, gives the results of eight years experience in the use of this medicine in private and dispensary practice, both in chronic and acute affections. In ten cases of goitre, it cured all in periods ranging from two weeks to two months, used internally and externally. Cases were treated at the same time with iodine; the treatment occupied the same length of time, and the cures were no more permanent or decided in the latter than in the former. He was led to give it a trial in whooping cough, upon the theory entertained by some pathologists, that there is a morbid condition and enlargement of the bronchial glands, or an irritating glairy mucus causative of the disease. In thirty-three cases of more than ordinary severity, ten in private, and the remainder in dispensary practice, its remedial influence was immediate and decided; the expectoration became bland, and less tenacious; paroxysms milder, and less frequent, and of shorter duration. He was better pleased with the result, than that of any remedy with which he is acquainted.

The medicine was given in doses of from one to five grs., according to age, and the duration of treatment averaged twenty days, but in the cases most favorably circumstanced, it did not extend over a period of more than nine to fifteen; where there existed pneumonia or bronchial complications, he added to the mixture, antimony or ipecac. The only inconvenience to which it gave rise in any of the cases, was a slight mucous diarrahœa, which was easily controlled.

Scrofulous lymphatic glands, such as those of the cervical region, which are so intractable, yielded rapidly to the ammonia, after iodine and its preparations had failed.

In the scaly varieties of skin diseases, as psoriasis inveterata, after failure of arsenic and iodine, rapid cures were effected. He thinks the benefit most marked in cases of psora, occurring in persons of irregular and dissipated habits, or where it is complicated with enlarged liver.

A writer in the ninth number of *Braithwaite*, gives his experience with it in pleurisy, subacute inflammations, and congestive conditions of the mucous membranes. With the Germans, as I have said, it usurps the place of nit. potass. save in those cases where the febrile symptoms run high.

Fuller, in his work on rheumatism, speaks of it as a remedy of singular value in chronic rheumatism, and wonders why it has found so little favor with the profession. He says that it not only increases secretion, mucous and cutaneous, but im proves nutrition. With bark it produces marvellous results in cases where other remedies have failed. It is not in those cases where the periostum and joints are effected, that its virtues are most satisfactorily displayed, but in those where the disease is chiefly confined to the muscular system.

Dr. Ebden, in the *Indian Annals of Medical Sciences*, for April, 1854, states that he has found it is a valuable remedy in all neuralgic pains, as facial neuralgia, nervous headaches, toothaches, sciatica and neuralgic dysmenorrhœa.

Some writers have spoken favorably of its use in hepatitis, where suppuration having commenced, mercury becomes inadmissible.

Fountain reports a case of uterine neuralgia, where it gave immediate relief, in ʒss. doses.

Prof. Barrillier, of Toulon, reports administering it in 259 cases of nervous cephalalgia, with success in 202, in doses of fifteen grs. in mint water, repeated every half hour; relief was felt immediately, seldom requiring the third dose. To prove effectual, he says it should not be used immediately upon the advent of the paroxysm, but when it was at its height of intensity. He says that it is not for all forms of cephalalgia, and mentions the following conditions as the result of his experience and conclusions :

1. It dissipates almost constantly idiopathic migraine or hemicrania, consecutive to *too abundant* menstruation. Other writers refer to its valuable properties in this connection.

2. It is powerless in the headaches, dependent upon irregular or suppressed menstruation.

3. It is tolerably successful in cranial pains, dependent upon derangements of the stomach, and the cephalalgia frequent in women and feeble persons, under the influence of sudden change of the atmosphere, prolonged intellectual labor on mental emotions.

4. It operates beneficially in cephalalgia, consecutive to repeated paroxysms of intermittent fever, those which are observed during the decline of severe fever, and in the course of the irritative period of typhus.

Stillé, in his late work upon the materia medica, speaks of it as reported to be of great value in chronic gleet, and leucorrhœa, and also as having been resorted to successfully in the treatment of several cases of quartan ague, when other medicines had failed.

Its decided and positive influence on the mucous membrane, renders it valuable in old coughs, dependent upon gastric derangement.

Dr. Hamill, of this city, tells me he has found it invauablle in the pneumonia of drunkards.

I need not occupy your time, by giving extracts from our journals of the last twelve or twenty-four months, detailing the cases of facial neuralgia, where its use was followed by an immediate and lasting success.

Dr. Watson speaks of its reputed value in hemicrania, and this, with the reported cases to which I allude, led me to use it. In facial neuralgia in some of its forms, I know of no one article of the materia medica which has so invariably afforded relief as this, given in doses of from ten to fifteen grs., every half-hour. If it does not remove the pain after some half-dozen doses, it is not apt to prove of service, and some other remedy must be resorted to.

Some one has thought it most serviceable in those cases attended with redness and swelling about the seat of pain. I am not satisfied that it is so. I have used it in the thin, sallow and anæmic. Once or twice in the intermittent variety, once successfully, once it failed. In nervous headaches, I have as yet, had little experience with it, but that has been attended with results which will lead me to try it further.

Were it only of equal value with other of our drugs, in these cases, its cheapness should lead us to use it in our practice among the poor,—a few cents furnishing a requisite quantity for medical purposes, and if it is furnished by the druggist, as charity, it costs him little.

I have thought that one reason of its undeserved neglect, was in consequence of its pungency, but this is in a great measure overcome, if it is given largely diluted, as the dose mentioned in an ounce of water. Its taste reminds me more of rock-salt, as we call it, than anything I think of. When the patient is one who does not object to an extra charge, for the sake of his palate, I have found the following formula acceptable:

| | | |
|---|---|---|
| ℞—Mur. Ammonia, | - - - | ʒij. |
| Water, | - - - - - | ℥iv. |
| Syr. Tolu, | - - - - | ℥ij. |
| Sugar, | - - - - - - - | ʒij. |
| Ess. Wintergreen, | - - | ʒij. |

Dose, ℥ss., which contains ten grs.

The *modus operandi* of this medicine, is not understood. It seems gradually to unload the vessels, and convalesence takes place without any critical discharge. The tongue becomes clean, and its decided influence upon the mucous membranes cannot be questioned.

I think there can be no mistaking its medicinal value as an alterative in certain chronic affections, for all the writers cited, seem to agree strikingly as to the results flowing from their experience, In this paper, I have tried to avoid all reference of a loose and conjectural character, giving only what seems to have been the result of actual experience,—and I think that the facts herein recorded, satisfactorily prove that it is a positive and valuable medicine.

